# A NOX2/Egr-1/Fyn pathway delineates new targets for TKI-resistant malignancies

**DOI:** 10.18632/oncotarget.4604

**Published:** 2015-06-23

**Authors:** Mary E. Irwin, Blake P. Johnson, Roxsan Manshouri, Hesham M. Amin, Joya Chandra

**Affiliations:** ^1^ Department of Pediatrics Research, The University of Texas MD Anderson Cancer Center, Houston, TX, USA; ^2^ University of Texas Graduate School of Biomedical Sciences at Houston, TX, USA; ^3^ Department of Hematopathology, The University of Texas MD Anderson Cancer Center, Houston, TX, USA

**Keywords:** TKI-resistance, CML, NOX, Fyn, Egr-1

## Abstract

Tyrosine kinase inhibitors (TKI) have improved CML response rates, and some are effective against resistance-promoting point mutations in BCR-ABL1. However, in the absence of point mutations, resistance still occurs. Here, we identify a novel pathway mediating resistance which connects p47phox, the organizer subunit of NADPH oxidase-2 (NOX2), with early growth response-1 (Egr-1) and the Src family kinase Fyn. We found up-regulation of p47phox, Egr-1, and Fyn mRNA and protein using paired isogenic CML cell lines and mined data. Isolation of CD34^+^ cells and tissue microarray staining from blast crisis CML patients confirmed *in vivo* over-expression of components of this pathway. Knockdown studies revealed that p47phox modulated reactive oxygen species and Egr-1 expression, which, in turn, controlled Fyn expression. Interestingly, Fyn knockdown sensitized TKI-resistant cells to dasatinib, a dual BCR-ABL1/Src inhibitor. Egr-1 knockdown had similar effects, indicating the utility of targeting Fyn expression over activation. Pointedly, p47phox knockdown also restored TKI-sensitivity, indicating that targeting the NOX2 complex can overcome resistance. The NOX2/Egr-1/Fyn pathway was also conserved within TKI-resistant EGFRΔIII-expressing glioblastoma and patient-derived glioblastoma stem cells. Thus, our findings suggest that targeting the NOX2/Egr-1/Fyn pathway may have clinical implications within multiple cancer types; particularly where efficacy of TKI is compromised.

## INTRODUCTION

The advent of tyrosine kinase inhibitors (TKIs) directed against BCR-ABL1, the primary oncogene associated with chronic myeloid leukemia (CML), has dramatically increased patient survival rates [1, 2]. However, TKI-resistance is a looming clinical problem as the number of patients with detectable disease burden continues to grow [2]. This issue underscores a need for new approaches to treat refractory patients. Single point mutations in BCR-ABL1 are known determinants of TKI-sensitivity [3–6]. However, such mutation-dependent resistance is currently treatable by second and third generation TKI such as nilotinib and ponatinib. Treatment options for mutation-independent resistance remain limited. Mutation-independent resistance is characterized by amplification of downstream BCR-ABL1 signaling or parallel activation of signal transduction proteins including Src family kinases such as Fyn [7]. In addition, persistence of CML stem cells (LSC), which are less dependent on BCR-ABL1 rendering them less sensitive to TKI, are another example of point mutation-independent resistance [8]. As stem cell populations are commonly less sensitive to inhibition of oncogenic tyrosine kinases, the identification of novel targetable pathways within those populations is of vital importance.

TKI-resistance is not a phenomenon that is exclusive to CML. For example, in glioblastoma multiforme (GBM) TKIs have been tested that target the epidermal growth factor receptor (EGFR) [9]. GBMs comprise the most aggressive and common form of primary brain tumor, conferring the worst clinical prognosis [10]. Patients with GBM survive little more than one year past diagnosis. EGFR amplification and activating mutations, EGFRΔIII and R108K, are commonly detected genetic alterations in GBM [11–13]. The most common EGFR mutation, EGFRΔIII, arises from an in-frame omission of 801 bp encoding the extracellular domain, rendering a truncated, yet constitutively active, form of the receptor [14, 15]. Such alterations of EGFR have been identified as strong indicators of worse patient survival in GBM [16]. Enrichment of EGFRΔIII expression is seen in a population of highly resistant GBM-derived stem cells (GSCs) [17–22]. Therapeutic strategies targeting EGFR with small molecule targeted-TKI, such as lapatinib, have yielded limited clinical efficacy, particularly in the setting of EGFRΔIII mutation [11, 23]. Therefore, strategies designed to therapeutically target TKI- resistance mechanisms may provide hope in a myriad of cancer types.

Prior work has identified that the elevated production of reactive oxygen species (ROS) occurs in multiple cancer types. Particularly, expression of BCR-ABL1, as well as other oncogenes, is known to result in increased levels of ROS within cancer cells [24–26]. The primary source of ROS in the majority of cells is the mitochondria [27]. As such, the mitochondrial influence on ROS in cancer has been extensively studied [28–30]. We have, however, discovered that alternative sources of ROS, such as the NADPH oxidase (NOX) complex, contribute to the resistant phenotype, through activation of a novel pathway in TKI-resistant CML and GBM. Collectively, our data in two cell line models of mutation-independent TKI-resistance, as well as patient samples, demonstrate a novel pathway connecting NOX2 to the transcription factor early growth response 1 (Egr-1) and subsequently Fyn, highlighting new mediators of TKI-resistance with potential to eradicate cancer stem cells.

## RESULTS

### Non-mitochondrial ROS production controls proliferation of TKI-resistant CML

**Figure 1:**
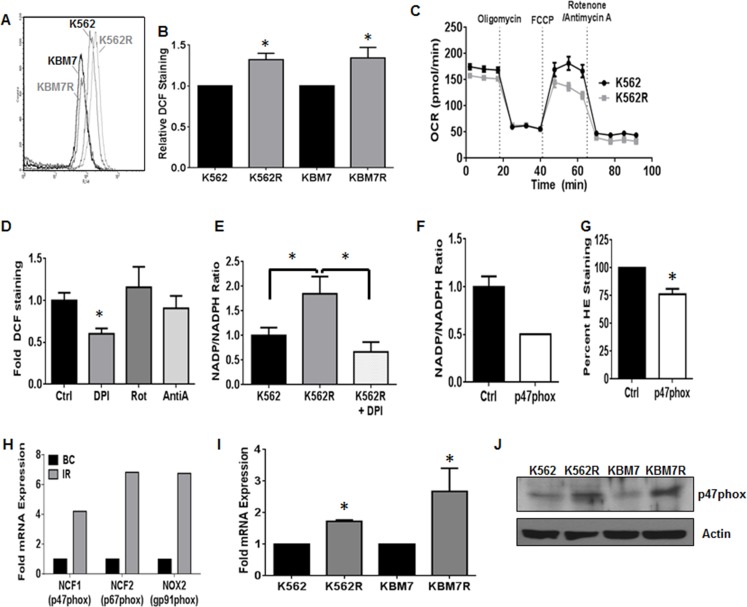
NOX2 promotes increased ROS in TKI-resistant CML TKI-sensitive (K562/KBM7) and resistant (K562R/KBM7R) cell lines were harvested and stained for ROS using DCF as described. A representative histogram is shown in **A.**, and staining quantified in **B.**. Bars are indicative of mean and SEM. * indicates *p* < 0.05. **C.** K562 and K562R cells were immobilized using Cell-Tak, and then oxygen consumption rates (OCR) measured over time with indicated treatments by Seahorse Bioanalyzer. All injections were 1 μM. **D.** Intracellular ROS levels were measured by flow cytometry using DCF staining as described after treatment with 30 μM DPI, 1 μM Rotenone, or 20 μM Antimycin A for 4 hours. Mean fluorescence intensity was normalized to control for each experiment. Bars indicate mean and SEM. * indicates *p* < 0.05 Unstained cells were utilized as a negative staining control. **E.** K562 (black bar) and K562R (grey bar) cells were plated at a density of 5×10^5^ cells and grown or treated with 30 μM diphenyleneiodonium (white spotted bar) for 4 hours. Cells were then lysed by freeze/thaw and lysates subjected to NOX activity assay as described. Bars indicate mean and SEM. * indicates *p* < 0.05. **F.** 72 hours post transfection with control (black bar) or p47phox (white bar) siRNA, NOX activity levels were measured in K562R cells as described. Bars indicate mean and SEM **G.** 72 hours post transfection with control (black bar) or p47phox (white bar) siRNA, superoxide levels were measured in K562R cells using HE staining as described. Mean fluorescence intensity was normalized to control for each experiment. Bars indicate mean and SEM. * indicates *p* < 0.05 Unstained cells were utilized as a negative staining control. **H.** Microarray data were mined [41] comparing TKI- resistant patients (IR, gray bar, *n* = 15) to blast crisis (BC, black bar, *n* = 28). Log (ratio) values were converted to ratios then normalized to blast crisis. **I.** TKI-sensitive (K562/KBM7) and -resistant (K562R/KBM7R) cell lines were harvested and cDNA made. qRTPCR was performed using p47phox directed primers. Bars indicate mean and SEM. * indicates *p* < 0.05. **J.** TKI-sensitive (K562/KBM7) and -resistant (K562R/KBM7R) cell lines were harvested and lysates subjected to SDS-PAGE followed by western blotting using p47phox and Actin antibodies. All data are representative of at least three individual experiments.

To understand how alterations of the redox milieu are associated with TKI-resistance in CML, we first measured the levels of ROS within two cell line models of acquired mutation-independent resistance. The TKI-sensitive/mutation-independent TKI-resistant pairs of CML cell lines (K562/K562R and KBM7/KBM7R; [31, 32]) were stained with dichlorofluorescein (DCF) to measure basal ROS expression (Figure 1A and 1B). ROS were significantly elevated in K562R and KBM7R cell lines as compared to their parental counterparts (Figure 1B). In order to more specifically pinpoint the source of ROS, we first analyzed the primary source of ROS in most cells: the mitochondria [27]. We measured oxygen consumption rates and mitochondrial respiration in both K562 and K562R cells. Mitochondrial respiration was similar between K562 and K562R cells (Figure 1C). Both cell lines did, however, display a basal level of non-mitochondrial respiration as evidenced by oxygen consumption in the presence of the mitochondrial complex I and III inhibitors, rotenone and antimycin A, suggesting potential alternative ROS sources. Thus, we utilized a variety of chemical ROS inhibitors to delineate a ROS source. While rotenone and antimycin A had no effect on ROS production of K562R cells (Figure 1D), diphenyleneiodonium (DPI) reduced ROS by 40% (*p* < 0.01). One of the potential targets of DPI is the NOX family of enzyme complexes. This enzyme family metabolizes NADPH to NADP^+^ converting oxygen to superoxide [27]. Interestingly, NOX activity was elevated 1.8-fold in K562R cells as compared to parental K562 cells (Figure 1E). DPI was sufficient to restore activity to baseline levels. Together, these data suggest that the primary source of elevated ROS levels in resistant CML is the NOX complex.

Recently, the NOX family has been described as a potential therapeutic target in CML [33–36]; however its contribution to the resistance phenotype remains unknown. CML cells have been noted to be particularly dependent on the NOX2 isoform which consists of NOX2, p67phox, p40phox, Rac1, and the key organizer subunit p47phox [27]. Knockdown of p47phox with siRNA resulted in a 50% reduction in NOX activity (Figure 1F) and an approximately 25% decrease in overall ROS levels of K562R cells (Figure 1G). Interestingly, mRNA expression of p47phox (NCF1), p67phox (NCF2), and gp91phox (NOX2) are up-regulated in samples from patients with resistance to imatinib as compared to blast crisis (BC) CML patients (Figure 1H). The same holds true in our isogenic model systems, where K562R and KBM7R cell lines have elevated mRNA and protein levels of p47phox compared to their parental counterparts (Figure 1I and 1J). Together, these data suggest that the NOX2 complex is expressed in, and controlling the redox milieu of, mutation-independent TKI-resistant CML.

### Fyn kinase is downstream of NOX2 in TKI-resistant CML

**Figure 2:**
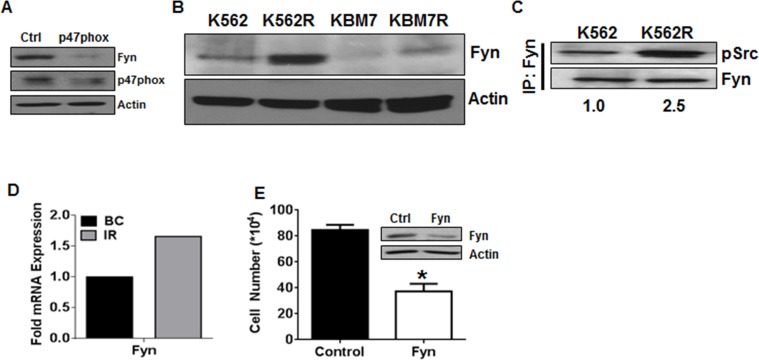
Fyn kinase is downstream of NOX2 in TKI-resistant cells **A.** 96 hours post transfection with control or p47phox siRNA, K562R cells were lysed and subjected to SDS-PAGE followed by immunoblotting for p47phox, Fyn, and Actin. **B.**K562, KBM7, K562R, and KBM7R cells were lysed and subjected to SDS-PAGE followed by immunoblotting for Fyn and Actin as a loading control. **C.** K562 and K562R cells were lysed then subjected to immunoprecipitation using antibodies directed against Fyn as described. Samples were washed in lysis buffer then boiled in loading dye prior to SDS-PAGE. Proteins were then immunoblotted using antibodies directed against pSrc Y416 and total Fyn. Densitometry was performed using ImageJ. **D.** Microarray data were mined [41] comparing Fyn mRNA expression between TKI-resistant patients (IR, gray bar, *n* = 15) to blast crisis (BC, black bar, *n* = 28). Log(ratio) values were converted to ratios then normalized to blast crisis. **E.** Viable cell number was counted 24 hours after nucleofection using siRNA directed against Fyn (white bar) or control siRNA (black bar) in K562R cells. Bars indicate mean viable cell yield and SEM. * indicates *p* < 0.05 Inset: Western blot of lysates after nucleofection with control or Fyn siRNA utilizing antibodies directed against Fyn or Actin. All data are representative of at least three individual experiments.

Our lab has previously established that the Src family kinase Fyn is controlled by the elevated ROS levels in CML [37], and overexpression of Fyn has been suggested as a mediator of imatinib resistance in CML [38–40]. However, the source of ROS and pathway leading to elevated Fyn are unknown. To determine if Fyn may be a downstream effector of NOX2-induced ROS, we analyzed Fyn expression after knockdown of p47phox (Figure 2A). Fyn was decreased when p47phox was knocked down, suggesting that Fyn is downstream of the NOX2 complex in mutation-independent TKI-resistant CML. To further associate Fyn with TKI-resistance in this setting, we probed for Fyn expression and activity in our cell line systems. Fyn was overexpressed in both TKI-resistant cell lines (K562R and KBM7R) as compared to their parental counterparts (Figure 2B). Additionally, Fyn activity, as measured by phosphorylation of Fyn, was elevated in K562R as compared to K562 cells (Figure 2C). These data are congruent with data from fifteen TKI-resistant patient samples where Fyn mRNA was up-regulated approximately 1.7-fold (Figure 2D) compared to BC samples [41]. Additionally, RNA-interference-based knockdown of Fyn decreased growth of K562R cells by 36% (Figure 2E) suggesting a functional role of Fyn in these TKI-resistant cells. Together, these data suggest that Fyn may be a downstream mediator of NOX2 effects in CML.

### Egr-1 is downstream of NOX2 and regulates Fyn expression in TKI-resistant CML

**Figure 3:**
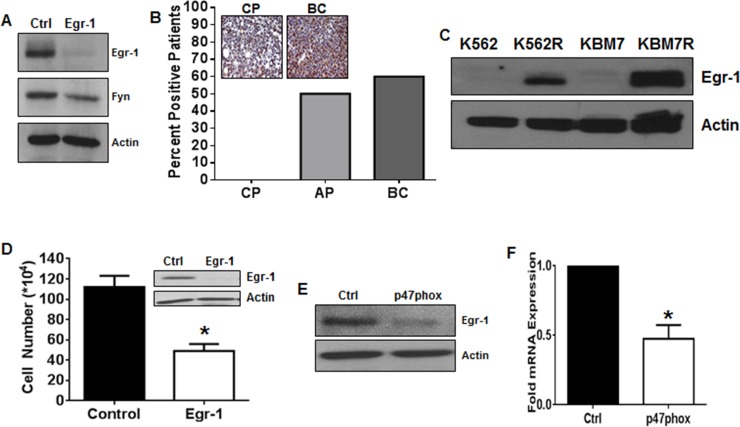
Egr-1 is the transcription factor connecting NOX2 to Fyn in TKI-resistant CML **A.** K562R cells were treated with control or Egr-1 directed siRNA for 24 hours then harvested. Lysates were subjected to SDS-PAGE followed by immunoblotting for Egr-1, Fyn, and Actin. **B.** Tissue microarrays containing samples from chronic phase (CP; *n* = 10), accelerated phase (AP; *n* = 6) and blast crisis (BC; *n* = 10) patients were probed with antibodies directed against Egr-1 then scored by a pathologist. The percent positive patients were calculated compared to negative and positive staining controls as described. **C.** Lysates from K562/K562R and KBM7/KBM7R cells were subjected to SDS-PAGE followed by immunoblotting with antibodies directed against Egr-1 and Actin. **D.** Viable cell number was counted 24 hours after nucleofection using siRNA directed against Egr-1 (white bar) or control siRNA (black bar) in K562R cells. Bars indicate mean viable cell yield and SEM. Inset: Western blot of lysates post-nucleofection with control or Egr-1 siRNA utilizing antibodies directed against Egr-1 and Actin. **E.** 96 hours post-transfection with control or p47phox siRNA, K562R cells were lysed the subjected to SDS-PAGE followed by immunoblotting for Egr-1 and Actin. **F.** 96 hours post-transfection with control or p47phox siRNA, K562R cells were lysed then subjected to qRTPCR using primers against Egr-1. Bars indicate mean and SEM. * indicates *p* < 0.05. All data are representative of at least three individual experiments.

To further delineate a pathway between NOX2 and Fyn in TKI-resistant CML, we focused on Egr-1. We have previously shown that Egr-1 is a transcription factor driving ROS-dependent Fyn expression in TKI-sensitive CML cells [37] however, little is known about differential Egr-1 expression in phases of CML, or its role in TKI-resistant disease. Knockdown of Egr-1 was sufficient to decrease Fyn protein levels in K562R cells (Figure 3A), suggesting that regulation of Fyn by Egr-1 holds true in the resistance setting. Our previous work has shown that Fyn expression is high in a panel of BC patients relative to those in chronic or accelerated phase [42], therefore, we examined Egr-1 protein expression in a tissue microarray (TMA) containing samples from CML patients in chronic phase (CP; *n* = 10), accelerated phase (AP; *n* = 6), and BC (*n* = 10) (Figure 3B). CP samples were exclusively negative for Egr-1 protein staining. However as CML progressed to AP and then to BC, 50% and 60% of patient samples were positive, respectively. Western blotting confirmed a four-fold overexpression of Egr-1 protein in BC samples compared to CP (data not shown). K562R and KMB7R cells were also analyzed for expression of Egr-1 (Figure 3C) which was increased in both cell lines compared with parental controls. Much like genetic inhibition of Fyn, knockdown of Egr-1 using siRNA decreased proliferation of viable K562R cells by 56% at 24 hours (Figure 3D). To determine if Egr-1 was indeed downstream of p47phox in K562R cells, we performed knockdown of p47phox using siRNA. When p47phox was depleted, Egr-1 was clearly decreased at both the protein (Figure 3E) and mRNA (Figure 3F) levels. Together these data suggest that a pathway exists in mutation-independent TKI-resistant CML whereby NOX2 induces Egr-1 expression leading to Fyn expression.

### Targeting the NOX2/Egr-1/Fyn pathway sensitizes resistant CML cells to TKI

**Figure 4:**
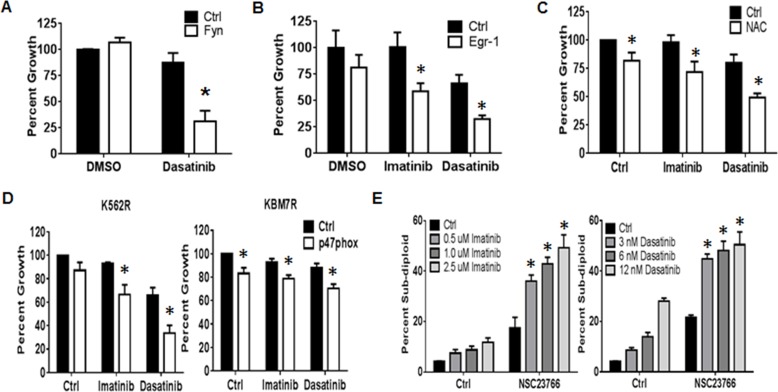
The NOX2/Egr-1/Fyn pathway controls TKI-resistance in CML **A.** K562R cells were nucleofected with siRNA directed against Fyn (white bars) or control siRNA (black bars). At 24 hours, cells were re-plated then treated with DMSO or 6 nM dasatinib for an additional 48 hours. Bars indicate mean percent growth and SEM. * indicates *p* < 0.05. **B.** Twenty four hours post nucleofection with control (black bar) or Egr-1-directed siRNA (white bar), K562R cells were re-plated and treated with 500 nM imatinib or 6 nM dasatinib for an additional 48 hours. Cells were then counted by ViCell. Bars indicate mean percent growth and SEM. * indicates *p* < 0.05. **C.** K562R cells were plated then pre-treated for 30 min with 24 mM NAC followed by 48 hour incubation with 500 nM Imatinib or 6 nM Dasatinib. Viable cell number was then counted by ViCell using trypan blue staining. Percent growth was calculated compared to untreated control. Bars indicate mean and SEM. * indicates *p* < 0.05. **D.** Seventy-two hours post transfection with control (black bar) or p47phox-directed siRNA (white bar), K562R and KBM7R cells were re-plated and treated with 500 nM imatinib or 6 nM dasatinib for an additional 48 hours. Cells were then counted by ViCell. Bars indicate mean and SEM. * indicates *p* < 0.05. **E.** K562R cells were treated with 250 μM NSC23766 alone or in combination with imatinib (doses as shown, left panel) or dasatinib (doses as shown, right panel). Cells were then stained with propidium iodide and analyzed by flow cytometry on the FL-3 channel. The percent sub-diploid was then measured. Bars indicate mean and SEM. * indicates *p* < 0.05. All data are representative of at least three individual experiments.

Thus far, we have shown that a pathway exists with the potential to alter proliferation of TKI-resistant CML. To determine the therapeutic relevance of targeting the NOX2/Egr-1/Fyn pathway in TKI-resistant CML, we first explored Fyn inhibition. Fyn kinase activity is effectively blocked by the second generation dual BCR-ABL1/SRC kinase inhibitor dasatinib. However, K562R cells are resistant to dasatinib, despite their high level of Fyn protein expression and activity (Figure 2B and 2C). RNAi-mediated knockdown of Fyn protein, however, was sufficient to significantly sensitize K562R cells to dasatinib by 56% (Figure 4A, *p* < 0.05). These data suggest that targeting Fyn kinase activity is inadequate for eliminating TKI-resistant CML and that targeting Fyn transcript is a potentially more effective method. Thus, we went up-stream in the pathway. Targeting Egr-1 with siRNA resulted in increased sensitivity of K562R cells to both imatinib (56%) and dasatinib (34%, Figure 4B, *p* < 0.05). However, there are no currently available therapeutic agents to target Egr-1. Egr-1 is well known as a redox sensitive transcription factor, and is decreased by treatment with the anti-oxidant N-acetyl cysteine (NAC, data not shown). We therefore determined if modulating the redox environment with NAC could also promote sensitivity. Indeed, NAC treatment was sufficient to reduce resistance to both imatinib (26%) and dasatinib (30%) in K562R cells (Figure 4C, *p* < 0.05). To determine if specifically targeting the NOX2 complex may be a viable option for TKI-resistant CML we used two methods. First, we performed knockdown of p47phox which significantly increased sensitivity to imatinib and dasatinib in both K562R and KBM7R cell lines (Figure 4D, *p* < 0.05). To confirm these results, we also treated K562R cells with NSC23766, an inhibitor of Rac1/2, another component of the NOX2 complex. Co-treatment of K562R cells with NSC23766 and TKIs resulted in significant induction of DNA-fragmentation compared to either treatment alone (Figure 4E, *p* < 0.05). Together, these results suggest that targeting the NOX2/Egr-1/Fyn pathway is a viable option for overcoming mutation-independent TKI-resistance in CML.

### The NOX2/Egr-1/Fyn pathway is active in EGFR-expressing GBM

**Figure 5:**
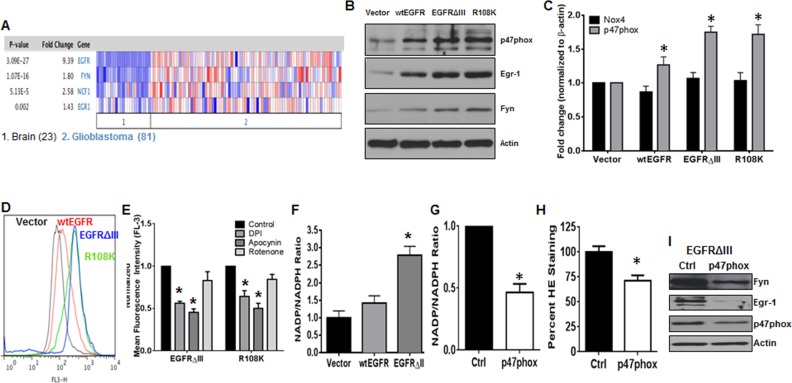
The NOX2/Egr-1/Fyn pathway is active in EGFR-mutated GBM **A.** Data were mined from Sun and colleagues [75] using Oncomine. The number 1 indicates normal brain tissues and the number 2 indicates GBMs. **B.** The U87 cell line model system expressing vector, wtEGFR, EGFRΔIII, or R108K-EGFR (R108K) were lysed then subjected to SDS-PAGE followed by immunoblotting for p47phox, Egr-1, Fyn, and Actin. **C.** The panel of U87 cell lines were lysed then subjected to qRTPCR using primers directed against NOX4 or p47phox. Bars indicate mean and SEM. * indicates *p* < 0.01. **D.** U87-vector, wtEGFR, EGFRΔIII or R108K cell lines were stained with HE then analyzed by flow cytometry on the FL-3 channel. Representative histograms are displayed. **E.** EGFRΔIII or R108K expressing U87 cell lines were treated with control, DPI (5 μM; 4 h), apocynin (100 μM; 24 h) or rotenone (1 μM; 4 h). Following treatment, intracellular ROS levels were measured by HE staining then analyzed by flow cytometry. **F.** NOX activity was measured in vector, wtEGFR, and EGFRΔIII-expressing U87 cells as described. Bars indicate mean and SEM. * indicates *p* < 0.05. U87-EGFRΔIII cells were transfected with control (black bar) or p47phox (white bar) siRNA. Forty-eight hours post-transfection, **G.** NOX activity and **H.** ROS levels were measured as described in materials and methods. Bars indicate mean and SEM. * indicates *p* < 0.05. **I.** U87-EGFRΔIII cells were transfected with either control or p47phox siRNA then harvested at 48h and subjected to SDS-PAGE followed by immunoblotting for p47phox, Egr-1, Fyn, and Actin. All data are representative of at least three individual experiments.

As mentioned previously, TKI-resistance is not a phenomenon that is exclusive to CML. Thus, to determine if the NOX2/Egr-1/Fyn pathway was active in another kinase-driven cancer type, we first mined data from two independent data sets. Both Sun and colleagues (Figure 5A) and the TCGA database (data not shown) show statistically significant increases in expression of p47phox (2.58-fold, *p* < 0.001), Egr-1 (1.43-fold, *p* < 0.01), and Fyn (1.8-fold, *p* < 0.001) mRNA, particularly in GBM where EGFR is also amplified. These data suggest that this pathway may also be active in EGFR-expressing GBM. To model this cancer type, we used the U87 glioblastoma cell line expressing either vector, wild-type EGFR (wtEGFR), EGFRΔIII, or EGFR with the R108K mutation (R108K). These cell lines were first probed for expression of p47phox, Egr-1, and Fyn by western blotting (Figure 5B). When EGFR was overexpressed and highly active, protein levels of all three components of this pathway were significantly increased. Much like in CML, the NOX family of enzymes has been described as a potential therapeutic target in GBM [43–45]. Of the seven family members, NOX4 has been noted as a prevalent isoform in GBM [43–45]. However, our data suggest that NOX2 may be the predominant isoform in EGFR-expressing GBM. We therefore performed qRTPCR for NOX4 and p47phox in our model system (Figure 5C). While p47phox mRNA was significantly up-regulated (1.3-fold in wtEGFR, 1.75-fold in EGFRΔIII, and 1.72-fold in R108K) by the presence of active EGFR (*p* < 0.01), there was no increase in NOX4. Much like in the CML setting, the more TKI-resistant EGFR-overexpressing cells had elevated levels of ROS (Figure 5D). To confirm the influence of NOX2 in GBM with active EGFR, we performed chemical inhibition of the NOX complex. The inhibitors DPI and apocynin were sufficient to decrease ROS levels of EGFRΔIII and R108K cell lines by 45% and 55% in EGFRΔIII and 36% and 50% in R108K, respectively, whereas rotenone did not significantly alter ROS in these settings (Figure 5E, *p* < 0.05). NOX activity was also up-regulated 2.8-fold in EGFRΔIII cells (Figure 5F, *p* < 0.05). Both heightened NOX activity (53%) and ROS (29%) levels were significantly quenched by siRNA-mediated knockdown of p47phox (Figure 5G and 5H, *p* < 0.05). Knockdown also revealed that the NOX2/Egr-1/Fyn pathway was intact in EGFRΔIII cells (Figure 5I). Together, these data suggest that the NOX2/Egr-1/Fyn pathway is present in multiple cancer types where it may promote a TKI-resistance phenotype.

### Effectors of the NOX2/Egr-1/Fyn pathway are potential targets in cancer stem cells

**Figure 6:**
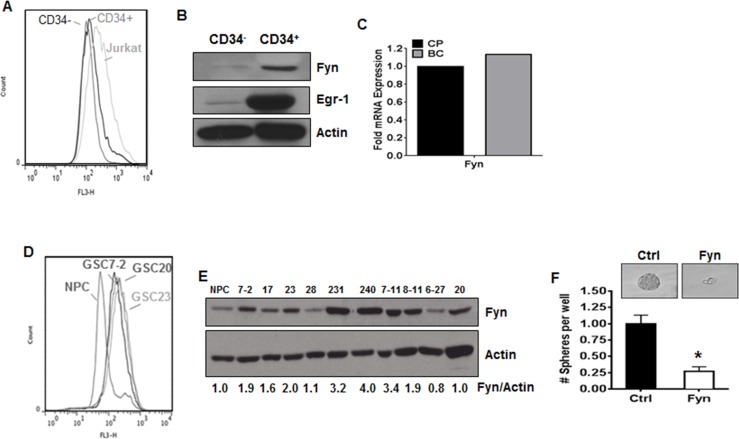
The NOX2/Egr-1/Fyn pathway is present in cancer stem cells **A.** CD34^+^ and CD34^−^ PBMCs were isolated as described and together with Jurkat cells (as a positive control) were stained with DCF. Representative histograms are shown. **B.** CD34^+^ and CD34^−^ PBMCs were isolated from a patient as described. Lysates were subjected to SDS-PAGE followed by immunoblotting for Fyn, Egr-1, or Actin. **C.** Microarray data were mined [48] comparing Fyn mRNA expression between LSCs from patients with chronic phase CML (CP, black bar) and blast crisis CML (BC, grey bar). **D.** Patient derived normal progenitor (NPC) or glioblastoma stem cell (GSC) lines were stained with HE then analyzed by flow cytometry on the FL-3 channel. Representative histograms are displayed. **E.** Glioblastoma stem cell (GSC) lines were lysed then subjected to SDS-PAGE followed by immunoblotting for Fyn and Actin. Densitometry was performed using ImageJ. **F.** GSC line 7-2 was transfected with control or Fyn siRNA and sphere-forming capacity was measured as described in materials and methods. Images are shown at 20x magnification. Bars indicate mean and SEM. * indicates *p* < 0.05.

TKI-resistance has been attributed to the persistence of cancer stem cells [8]. These stem cell populations more closely recapitulate properties observed within human primary disease and tend to be the most highly resistant to TKI [8] because they are less dependent on BCR-ABL1 [46]. We therefore sought to determine if LSCs derived from CML patients had altered levels of ROS. BC-CML is associated with increased CD34^+^ cells [47]. We isolated CD34^+^ cells containing LSCs from a CML patient in BC and determined ROS levels compared to CD34^−^ cells. Indeed, ROS were elevated in CD34^+^ cells containing LSCs isolated from BC patients as compared to CD34^−^ cells (Figure 6A). To determine if the downstream effectors of this pathway were present in LSC containing populations, we performed western blotting for Egr-1 and Fyn. The expression of Egr-1 was up-regulated 5.4-fold and Fyn was elevated 3.9-fold in CD34^+^ cells compared to their CD34^−^ counterparts (Figure 6B). Additionally, microarray data from BC-LSCs, which are potentially more treatment resistant than CP-LSCs, showed up-regulation of Fyn mRNA (Figure 6C). As shown in figure 5, the NOX2/Egr-1/Fyn pathway is a common signature of resistance within a subset of GBM. Thus, to determine if this pathway was also relevant in GSCs, ROS was measured in a panel of patient-derived GSCs. Much like LSCs, ROS levels were elevated in GSCs as compared to normal progenitor cells (Figure 6D). Fyn expression, as evaluated by western blotting, was increased in eight of ten GSC cell lines (Figure 6E). Knockdown of Fyn, specifically in the GSC7-2 line, resulted in a 75% attenuation of sphere forming capacity (Figure 6F). Together, these data suggest that the NOX2/Egr-1/Fyn pathway may be a novel therapeutic target for the eradication of the more treatment resistant cancer stem cell populations.

### Many potential novel targets are downstream of the NOX2/Egr-1 pathway

**Figure 7:**
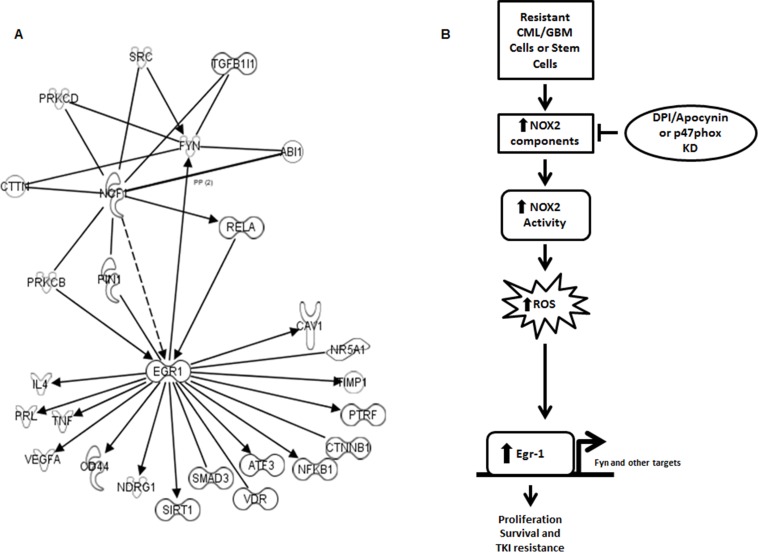
A number of pathways link NOX to Egr-1 and subsequent proliferation and survival **A.** Ingenuity pathway analysis was utilized to compare the potential linking pathways between NCF1 (p47phox) and Egr-1/Fyn. Potential linking targets are those that show congruent regulation and interaction in TKI-resistant cell lines and LSCs from blast crisis patients over chronic phase LSCs [39, 48]. **B.** Our working model predicts that resistant CML and GBM cells share a pathway through which an oncogene promotes ROS generation through NOX2 leading to Egr-1 expression leading to proliferation and survival even in the presence of TKIs.

To determine potential mediators of NOX2-induced Egr-1 expression and ultimately TKI-resistance, we utilized ingenuity pathway analysis to uncover genes that were coordinately regulated in TKI-resistant CML [39] and BC-LSCs [48] (Figure 7A). Potential mediators of the NOX2–Egr-1 pathway include PKCβ, PIN1, and RelA. Additionally, multiple downstream mediators of proliferation and survival were coordinately up-regulated downstream of Egr-1 which include a myriad of proteins in addition to Fyn (Figure 7A). Collectively, our data highlight the NOX2/Egr-1/Fyn pathway as viable options for overcoming mutation-independent acquired TKI-resistance.

## DISCUSSION

Our data indicate that a novel pathway is present in TKI-resistant CML, and EGFRΔIII GBM, cells whereby NOX2 is elevated and activated leading to increased levels of ROS and subsequent Egr-1 protein expression resulting in proliferation and survival of resistant cells (Figure 7B). This novel pathway can be inhibited by specific targeting of the NOX complex, ROS scavenging, or direct inhibition of Egr-1 and its downstream effectors. Thus, our data delineate new options for the therapeutic targeting of CML in patients with mutation-independent TKI-resistance, with the potential for similar effects in other TKI-resistant cancer types.

The NOX family has been suggested as potential therapeutic targets in prostate and pancreatic cancers, as well as Burkitt's lymphoma, melanomas, glioblastomas and TKI-sensitive CML [33–36, 49–52]. Of the seven NOX complex family members, NOX1, NOX2, NOX4, NOX5, and DUOX2 have all been identified as isoforms present in CML [27, 33, 35]. Specifically, Sanchez-Sanchez and colleagues have shown expression of NOX2, NOX5, and DUOX2 in K562 cells and CML patient samples. In this setting, NOX2 was the most highly expressed isoform. Additionally, they combined use of BCR-ABL1-directed TKI and the flavo-enzyme inhibitor DPI which resulted in synergistic cell death of TKI-sensitive K562 cells *in vitro*. Further, this combination was effective in two independent mouse models of TKI-sensitive CML, and inhibited proliferation of CD34^+^ cells isolated from CML patients [33]. However, this study failed to determine the contribution of NOX to mutation-independent TKI-resistant CML. Similarly, Landry and colleagues found that targeting p22phox, a component of NOX1, NOX2, and NOX4, promoted cell death of TKI-sensitive CML cells [35]. However, much like the previous study, the role of the NOX complex in TKI-resistance was not established. We have found that p47phox, an organizer subunit found within the assembled NOX2 complex, is significantly up-regulated in two mutation-independent TKI-resistant CML cell lines and a TKI-resistant patient population (Figure 1H–1J). Specifically, data mined from Radich and colleagues noted that, while the majority of genes from chronic phase patients with relapse after imatinib treatment looked similar to that of advanced phase (BC) patients [41], other genes, such as the NOX2-related genes shown here, were uniquely altered in patients with TKI-resistance, suggesting potential roles for these molecules in the resistance phenotype. However, the mutational-dependence of the resistance was not well described as the patient data was not stratified by mutation status. We further show that the NOX2 complex controls elevation of ROS in resistant cells, since targeting p47phox with siRNA was sufficient to decrease both NOX activity and ROS in K562R cells (Figure 1F and 1G). Utilizing two methods to target the NOX2 complex (p47phox knockdown and the Rac1/2 inhibitor NSC23766) we found that inhibition of NOX2 was sufficient to increase sensitivity to BCR-ABL-directed TKI (Figure 4E and 4F). Thus, NOX2 may be a therapeutically relevant target in both TKI-sensitive and -resistant CML.

We and others have identified the Src family kinase Fyn as a potential redox-dependent mediator of TKI-resistance in CML [38–40, 42]. Fyn is indeed up-regulated in TKI-resistant CML cell lines (Figure 2B) however inhibition of Fyn kinase activity is insufficient to overcome pan-TKI-resistance (Figure 4A). Interestingly, knockdown of Fyn re-sensitizes cells that are resistant even to Fyn kinase inhibition by dasatinib (Figure 4A) suggesting that targeting Fyn protein itself may be a better therapeutic option. We have found that Fyn is downstream of p47phox (Figure 2A), and Egr-1, the transcription factor known to regulate redox-sensitive Fyn expression [37], is an intermediary of this pathway (Figure 3A, 3E and 3F). Consistent with our previous studies in TKI-sensitive CML [37], knockdown of Egr-1 resulted in decreased Fyn protein expression (Figure 3A), and much like Fyn [42], Egr-1 protein was increased as patients progressed from CP to BC CML (Figure 3B). Two cell line models of mutation-independent TKI-resistance (K562R and KBM7R; Figure 3C) had elevated Egr-1 protein expression. Egr-1 itself has varying roles in cancer depending on the cellular context. In breast cancer, fibrosarcoma, and MDS, Egr-1 has been described as a tumor suppressor [53–56]. However, in ovarian cancer, ALK^+^ lung adenocarcinoma, and prostate cancer, Egr-1 is oncogenic [57–62]. Knockdown of Egr-1 in TKI-resistant CML cell lines was sufficient to decrease proliferation (Figure 3D) suggesting that Egr-1 plays an oncogenic role in CML. Further, decreasing Egr-1 expression by siRNA re-sensitized resistant cells to BCR-ABL1-directed TKI (Figure 4B), suggesting that Egr-1 may be a novel target for the control of mutation-independent TKI-resistance.

With their relative success in CML, TKIs which target other oncogenic tyrosine kinases have been tested in other cancers, such as lapatinib in EGFR-expressing GBM [11–13]. However such strategies have yielded only limited clinical efficacy [11, 23]. We found that components of the novel NOX2/Egr-1/Fyn pathway were also commonly up-regulated in EGFR-expressing patient GBM patient samples (Figure 5A) and cell line models (Figure 5B and 5C), where they remained dependent on p47phox (Figure 5I). Further, while NOX4 has been the primary NOX isoform associated with GBM, we have found that GBM with active EGFR over-expresses NOX2, which results in elevated ROS levels (Figure 5C–5H). Together, these data indicate that the NOX2/Egr-1/Fyn pathway is also pertinent to other TKI-resistant cancer types.

Cancer stem cells are being increasingly recognized as a significant contributor to relapsed and refractory disease, and in CML, elegant studies indicate that the LSC population is less reliant upon BCR-ABL1 for survival and therefore less susceptible to direct inhibition of the oncogenic kinase [8, 46]. We find that ROS levels are heightened in LSCs from BC patients (Figure 6A), which is consistent with reports of increased ROS and increased oxidative phosphorylation in CML LSCs [63, 64]. Increased Egr-1 and Fyn are seen in this population (Figure 6B and 6C) indicating that components of this ROS dependent pathway are overexpressed in LSCs. These data extend to other cancer stem cell models, since ROS were similarly elevated in patient-derived GSC cell lines (Figure 6D), and the endpoint of this pathway, Fyn, was also overexpressed (Figure 6E). Importantly, knockdown of Fyn protein was sufficient to decrease GSC sphere formation (Figure 6F). Together, these data suggest that the NOX2/Egr-1/Fyn pathway may also have potential for the eradication of cancer stem cells across cancer types.

While the pathway through which the NOX2 complex elevates Egr-1 expression remains unknown, ingenuity pathway analysis of TKI-resistant cell lines and blast crisis LSCs reveal a number of different pathways through which this might occur including NFκB, PIN1, or PRKCB (Figure 7A). These pathways are the subject of future experiments. Additionally, Egr-1 may have a myriad of targets in addition to Fyn through which it may promote resistance. Indeed, sixteen potential targets that mediate proliferation and survival are downstream of Egr-1 and are coordinately regulated in resistant cells and LSCs (Figure 7A). Of these, four targets are already noted to associate with TKI-resistance. For example, CD44, a cell surface glycoprotein involved in cell adhesion and migration, and SIRT1, an NAD-dependent deacetylase, are elevated in imatinib resistant samples [39, 65]. Also inhibition of the NFκB pathway can overcome imatinib resistance [66]. Our pathway analysis suggests therefore that NFκB may be a mediator of the pathway, and a target of its action (Figure 7A). Additional targets include beta-catenin signaling, which is known to protect LSCs from TKIs [67], as well as TNF and VEGFA, which may be involved in other types of treatment resistance in CML [68, 69]. The remaining ten targets have yet to be studied in the context of treatment resistance in CML (Figure 7A). Thus, our data suggest a number of potential novel targets downstream of Egr-1 that may contribute to TKI-resistance in CML that will be the subject of future studies.

Together, our findings suggest that the NOX2/Egr-1/Fyn signaling axis may represent a novel target in TKI-resistant cancers, particularly in a population of treatment-refractory stem cells. Unfortunately, as a transcription factor, Egr-1 is difficult to specifically target with therapeutics. Also, though the classic NOX inhibitors such as the ones employed in this study (apocynin and DPI) lower NOX activity, they display a non-specific pattern of NOX-targeting, consequently limiting their clinical utility [70]. However, more specific NOX2 inhibitors are currently under evaluation including the p47phox binding peptide, NOX2ds-tat [71], providing promise for the clinical application of NOX2-targeted strategies. Collectively, these findings indicate that targeting NOX2 activation via inhibition of p47phox represents a plausible means of targeting TKI-resistant malignancies.

## MATERIALS AND METHODS

### Chemicals and cell lines

Imatinib and dasatinib were purchased from LC Laboratories (Woburn, MA). DPI, apocynin, rotenone, and NAC were purchased from Sigma-Aldrich (St. Louis, MO). Egr-1 siRNA was purchased from ThermoScientific (Pittsburgh, PA) and p47phox and Fyn siRNA from Santa Cruz Biotechnology (Dallas, TX). K562 cells, originally isolated from pleural effusion of a 53 year old female with BC-CML and are granulocytic in nature [72], were obtained from American Type Culture Collection (Manassas, VA) and maintained in RPMI1640 with 10% FBS, 1% L-glutamine, and 1% penicillin/streptomycin. K562R cells, previously confirmed to be TKI-resistant without BCR-ABL1 mutation, were kindly provided by Drs. Ellen Weisberg and James D. Griffin [32] and were cultured in RPMI1640 with 10% FBS, 1% L-glutamine, and 1% penicillin/streptomycin containing 0.5 μM imatinib. KBM7 cells were isolated from a 39 year old male in BC and are near haploid [73]. KBM7 and KMB7R (previously found to be TKI-resistant without mutation of BCR-ABL1) cell lines were kindly provided by Dr. Zeev Estrov [31, 73] and were cultured in IMDM with 10% FBS, 1% L-glutamine, and 1% penicillin/streptomycin. The human GBM cell line U87-MG stably overexpressing wild-type EGFR, EGFRΔIII or EGFR-R108K was cultured in DMEM/F12 containing 10% FBS, 2 mM glutamine, 100 U/ml penicillin, 100 mg/ml streptomycin. The cells were routinely supplemented with 50 μg/ml of Zeocin (Life Technologies, Carlsbad, CA). Patient-derived glioblastoma stem cells (GSCs) [74] were provided by Drs. Frederick Lang and Erik Sulman and were cultured in DMEM/F12 containing 20 ng/mL EGF (Sigma-Aldrich) and bFGF (Life Technologies). All cell lines were cultured in 5% CO_2_ at 37°C.

### Assessment of the redox environment

Reactive oxygen species were measured by staining with DCF or dihydroethidium (HE). Briefly, equal cell numbers were washed with PBS then stained with DCF or HE (Life Technologies) in 1X PBS for 30 min at 37°C in the dark. Samples were centrifuged and re-suspended in PBS then analyzed on the FL-1 or FL-3 channel of a flow cytometer, respectively. Mean fluorescence of 10,000 cells was then normalized to controls for each experiment. To measure mitochondrial respiration, 8 × 10^5^ K562 or K562R cells were plated into seahorse 96-well plate using Cell-Tak (BD Biosciences, San Jose, CA). Mitochondrial respiration was then measured using the XF Cell Mito Stress Test Kit and XF^e^ extracellular flux analyzer (Seahorse Biosciences, North Billerica, MA). All injections were 1 μM. NADPH oxidase activity was measured using an NADP^+^/NADPH assay kit (Abcam) per manufacturer's instructions. Briefly, 1.5 × 10^6^ K562 or K562R cells, or 2.0 × 10^5^ cells/mL GBM cells, were lysed by freeze/thaw in NADPH extraction buffer. To detect NADP^+^, samples were decomposed by heating as per manufacturer's instructions. Samples were loaded onto 96-well plate and compared to standard curve as per instructions.

### Data mining

Data were mined from Radich and colleagues for Fyn, NCF1, NCF2, and NOX2 expression in TKI-resistant patients (*n* = 15) and normalized to expression of blast crisis patients (*n* = 28) [41]. TKI-resistant patients were patients who relapsed after initial treatment response, presented again with chronic phase disease, and had various BCR-ABL1 mutational statuses. These data could not be sub-divided by mutational status as they were presented as an averaged value. The Oncomine database was queried to identify alterations occurring in gene expression. Oncomine 4.5 database analysis tool is available with a subscription at http://www.oncomine.org. For GBM, selected data [75] was compared for gene expression levels in primary glioblastoma tumor samples relative to normal brain controls. For ingenuity pathway analysis, pathway connections between Egr-1, Fyn, and p47phox were analyzed in imatinib resistant cell lines compared to parental cells [39]. These targets were then compared to targets in LSCs from BC patients compared to CP [48].

### Real-time PCR

Total RNA was purified using an RNeasy Mini Kit (QIAGEN, Valencia, CA). Reverse transcription reaction was performed for each sample (1 μg of RNA) via iScript RT kit (Bio-Rad) per the manufacturer's protocol. Real-time PCR was carried using the iTaq Universal SYBR Green PCR master mix in a 20 μL total volume. The PCR primer sequences and conditions for human Fyn [37], Egr-1 [76], NOX-4 [45] and p47phox [45] were previously described. Relative gene expression was calculated by determination of the cycle threshold (C_t_) value and normalizing to actin C_t_ values.

### Detection of protein

Cells were lysed in triton lysis buffer (PBS with 1% Triton X-100; 25 mM Tris, pH 7.5; and 150 mM NaCl) containing protease inhibitor cocktail (Roche, Indianapolis, IN) for 1 hour at 4°C. Lysates were then cleared by centrifugation and protein abundance assessed by Bradford assay (Bio-Rad, Hercules, CA). Fifty micrograms of sample was added to laemelli buffer then boiled for 5 minutes. Lysates were then subjected to SDS-PAGE followed by transfer to PVDF membrane (Bio-Rad). Membranes were blocked for 1 hour in 5% milk and then exposed to Fyn (Cell Signaling Technology, Danvers, MA), Egr-1 (Cell Signaling Technology), p47phox (Santa Cruz Biotechnology), or Actin (Sigma-Aldrich) antibodies overnight. Membranes were washed three times in tris buffered saline containing 1% triton (TBST) for 30 minutes followed by incubation with secondary antibodies (GE Healthcare, Pittsburgh, PA). Proteins were visualized with ECL (Bio-Rad) and densitometry was measured with NIH ImageJ (Bethesda, MA).

### Patient samples and immunohistochemistry

Patient specimens were collected after informed consent was obtained in accordance with the Declaration of Helsinki. TMA studies were initiated under approval from the University of Texas M. D. Anderson Cancer Center Institutional Review Board. Formalin-fixed and paraffin embedded (FFPE) tissue samples from bone marrow biopsies or clots were collected from CML patients in CP (*n* = 10), AP (*n* = 6) or BC (*n* = 10) and amassed in a TMA as previously described [34, 77]. Positive control (FFPE cell block prepared from K562 cells) and negative control (normal lung epithelium tissue) slides were stained at the same time with the TMA. Slides were deparaffinized and rehydrated then subjected to antigen retrieval for 2.5 min. Thereafter, slides were washed with PBS containing 0.05% Tween-20 (PBST), and 0.03% H_2_O_2_ was added for 15 min. Slides were then blocked for 30 min using serum-free protein block before incubation overnight at 4°C with anti-Egr-1 antibody (Ab54966; Abcam, Cambridge, MA) diluted 1:150 in 5% bovine serum albumin. Subsequently, detection was achieved by addition of the biotinylated secondary antibodies for 30 min, followed by streptavidin for 30 min. The slides were developed using 3,3′-diaminodbenzidine tetrahydrochloride substrate (DAB) kit (Dako, Carpinteria, CA) that includes horseradish peroxidase, and hematoxylin was used for counter staining. Microscopic evaluation was performed without knowledge of disease stage or clinical outcome. The percentage of positively stained cells was determined by counting at least 100 neoplastic cells in each of the TMA cores. Photomicrographs were obtained using a Nikon Microphot FXA microscope (Nikon Instruments, Melville, NY) and an Olympus DP70 camera (Olympus America, Melville, NY).

For western blots, samples from CP, AP, and BC bone marrow and peripheral blood were obtained and frozen in liquid nitrogen. Samples were then thawed and red blood cells were lysed by incubation in 1:1 PBS:ACK lysis buffer for 10 minutes on ice. Samples were centrifuged then lysed with lysis buffer as described earlier. For CD34^+^ isolation, peripheral blood mononuclear cells were transferred to MACS magnetic bead separation column for separation as per manufacturer's instructions (Miltenyi Biotec, San Diego, CA). CD34^−^ lysate was collected as flow through and CD34^+^ cells were collected from the column. Cells were then lysed and subjected to western blotting as described earlier.

### Knockdown experiments

Egr-1 or Fyn-directed siRNA was electroporated into K562R cells utilizing an Amaxa nucleofection machine (Lonza, Allendale, NJ) according to manufacturer's instructions. P47phox siRNA was transfected into all cell lines using lipofectamine RNAiMAX (Life Technologies) according to manufacturer's instructions. Knockdown was evaluated by western blotting as described earlier. After knockdown (24 hours for Fyn and Egr-1, 72 hours for p47phox), cells were treated with imatinib or dasatinib for 48 hours. Proliferation was then measured using hemocytometer counting with trypan blue staining or a ViCell Viability Analyzer (Beckman Coulter, Fullerton, CA).

### GSC sphere-formation assay

Sphere formation was performed as previously described [18]. Briefly, cells were dissociated and transfected with either control or Fyn siRNA (30 pmol) and then plated 24 hours later at a density of 5,000 cells per mL. Spheres > 50 μM were counted 10 days later.

### Statistical analyses

Unless otherwise stated, values listed in figures are expressed as the mean ± SEM of at least three replicates. Statistical comparisons were made using GraphPad Prism 4.0 software (GraphPad Software, Inc., La Jolla, CA) by Student's *t*-test. A *p*-value of < 0.05 was considered significant.
